# Ultrasonographic insights into the complex anatomy and biomechanical dynamics of the Achilles tendon and its fascicles: a pictorial essay

**DOI:** 10.1007/s40477-025-00987-z

**Published:** 2025-02-03

**Authors:** Stella Salvatore Massimo, Vita Fabio, Suhel Gabriele Al Khayyat, Donati Danilo, Ciampi Barbara, Becciolini Marco, Tedeschi Roberto, Faldini Cesare, Galletti Stefano

**Affiliations:** 1 Advanced Musculoskeletal Ultrasound, Department of Clinical and Experimental Medicine, SIUMB School of Pisa, Santa Chiara University Hospital, Pisa, Italy; 2 IRCCS Istituto Ortopedico Rizzoli, 1st Orthopaedics and Traumatology Clinic, Bologna, Italy; 3 Rheumatology Unit Department of Medical Sciences, Surgery and Neurosciences, University of Siena, Siena, Italy; 4 Physical Therapy and Rehabilitation Unit, Policlinico di Modena, Modena, Italy; 5 Clinical and Experimental Medicine PhD Program, University of Modena and Reggio Emilia, Via Università, 4, 41121 Modena, Italy; 6 Department of Biomedical and Neuromotor Sciences, Alma Mater Studiorum, University of Bologna, Bologna, Italy; 7 Musculoskeletal Ultrasound School, Italian Society for Ultrasound in Medicine and Biology, Bologna, Italy

**Keywords:** Achilles tendon, Ultrasound, Achilles tendon fasciculation, Tendinopathy, Retrocalcaneal bursa

## Abstract

This pictorial essay clarifies the complex anatomical and ultrasonographic features of the Achilles tendon. Utilizing high-frequency ultrasound probes (12–18 MHz), the study visualizes the tripartite segmentation of the tendon as it relates to anatomical fascicles. Notably, there is variability in the ultrasound visibility of fascicles and this partition influences biomechanical properties and susceptibility to injury. This essay not only enhances the understanding of Achilles tendon anatomy but also underscores the importance of anatomical knowledge for comprehending associated pathologies. The findings suggest potential areas for further research into tendon pathology, facilitating the development of targeted rehabilitation strategies.

**Supplementary Information:**

The online version contains supplementary material available at 10.1007/s40477-025-00987-z.

## Introduction

The Achilles tendon, also known as the Hippocratic 'chorda magna,' is the largest and strongest tendon in the human body. It serves as the common tendon of the gastrocnemius and soleus muscles, which together constitute the triceps surae muscle complex.

It represents a critical biomechanical structure in the human body, integrating complex structural and functional attributes.

This tendon is critical to the propulsion and stabilization phases of gait, influencing walking, running, and jumping activities [1]. Its robust yet flexible structure allows for the absorption and transmission of high force loads between the musculature and the heel bone. Anatomically, the Achilles tendon is the thickest and strongest tendon in the human body, typically measuring approximately 15 cm in length and 6 cm^2^ of cross-sectional area in adults.

Despite its strength, the tendon’s unique composition and high tensile load make it susceptible to both acute and chronic injuries, which are prevalent among athletes and the general population.

These injuries range from mild tendinitis to complete ruptures, with recovery often prolonged and complex.

The gastrocnemius muscle with its lateral and medial head, is a biarticular muscle and is inserted into the superior and posterior portion of the respective femoral condyles and partially also into the joint capsule (the lateral head may contain the fabella, an inconstant sesamoid) while the soleus is a monoarticular muscle, originating from the head at the lateral and posterior margin of the proximal portion of the fibula and from a fibrous arch that forms between the fibula and the posterior portion of the tibia, in its proximal third. The tendon is widest at the origin, gradually narrowing almost to the height of the dorsal portion of the tibiotarsal and then widening again at the level of the insertion on the posterior surface of the calcaneus [1, 2].

The microstructure of the Achilles tendon reveals a meticulously organized network of collagen fibers, which are grouped into fascicles. These fascicles are connected by the endotenon, with the entire structure enclosed within the epitenon and externally by the paratenon, both forming the peritenon, a connective sheath that envelops the Achilles tendon. This hierarchical arrangement is critical to the tendon's ability to withstand repetitive stress and adapt to dynamic mechanical demands. Therefore the tendon does not have a true synovial sheath, but instead relies on a peritenon to provide a double-layered (epitenon and paratenon) lubricating ‘sheath’ that facilitates movement within the surrounding tissues. Biomechanically, the Achilles tendon displays unique properties, functioning primarily to transmit mechanical force generated by the hind leg muscles to the heel, enabling plantar flexion of the foot and facilitating postural balance and locomotion. The viscoelastic characteristics of the tendon allow it to act as a spring, storing and releasing energy during activities such as running, thereby improving efficiency and reducing metabolic costs [3–10].

Given its critical role in daily mobility and high-performance sports, any compromise in its function can have a significant impact on an individual's quality of life. Advanced imaging techniques such as ultrasound (US) and magnetic resonance imaging have become indispensable tools in the anatomical and functional assessment of this tendon. These modalities not only aid in the diagnosis of the pathology but also provide valuable information about the morphological and biomechanical properties of the tendon in real time.

Understanding the normal and variant anatomy of the Achilles tendon through detailed imaging can guide clinicians in designing effective management strategies for various pathologies. This illustrated essay aims to clarify these issues through high-resolution US images, highlighting the complex anatomy of the tendon, its typical presentation and the potential clinical implications of various findings.

## US Anatomy

US examination with high-frequency linear probes (12–18 MHz) is necessary for tendon evaluation. With the patient in a prone position and foot off the bed, a distinct tripartite segmentation is typically visible in axial scans. This segmentation becomes especially clear during dynamic assessment, as the probe is slid from top to bottom to visualize the two superficial fascicles and a deeper central fascicle [4].

**Fig. 1:**
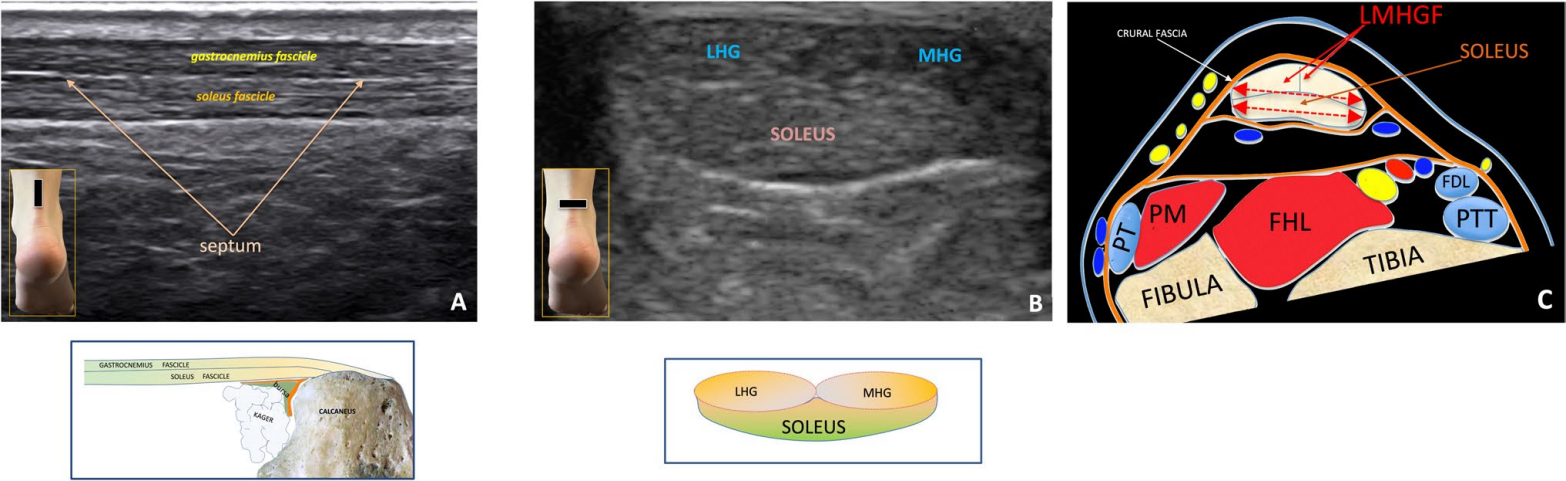
**A** Longitudinal scan of the Achilles tendon showing its two components: the superficial gastrocnemius fascicle, the deep soleus fascicle and the septum in the middle. Below the figure, a schematic illustration of the anatomical section is shown. **B** Axial scan of the Achilles tendon. Note the three components, two in the upper portion (gastrocnemius) and one in the lower part (soleus). The boundary between the two heads of the gastrocnemius appears indistinct on short-axis ultrasound, and no clear vertical septum is visible. The drawing under this figure illustrates the anatomical section. **C** Schematic anatomical transverse section of the Achilles tendon and its regional anatomical relationships. *LMHGF* Lateral and Medial Head of Gastrocnemius Fascicles; *FHL* Flexor Hallucis Longus muscle, *PT* Peroneal Tendons, *FDL* Flexor Digitorum Longus tendon, *PTT* Posterior Tibial Tendon

In a longitudinal scan, the tendon often appears bipartite, with a central thin hyperechoic septum separating the two components. In axial scans, tripartite fasciculations are observed but the border between the two heads of the gastrocnemius is faded. In short axis no vertical septum is clearly visible (Fig. 1).

**Fig. 2:**
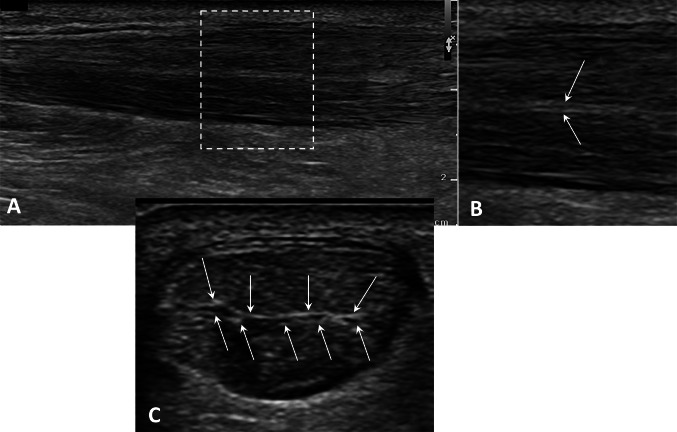
**A** In this case of moderate tendinopathy, the longitudinal scan of the Achilles tendon shows peritenon duplication of the septum, with the two fascicles maintaining their own peritenon. **B** Enlarged detail (in long-axis scan) of the dashed rectangular area shown in A. White arrows indicate the double peritenon. **C** The separation of the fascicles with peritenon duplication is also clearly visible in axial section. A small amount of effusion is visible between the two layers of the duplicated peritenon. White arrows indicate the double peritenon

In rare cases it is possible to observe a duplication of the peritenon of this septum: in this eventuality the two fascicles maintain their own peritenon. The separation of the fascicles with the duplication of the peritenon is clearly visible with US both in long axis than in axial section (Fig. 2).

These findings provide a significant improvement in our understanding of the fascicular arrangement of the tendon and its implications for both normal function and pathology.

### Fascicles visibility and echogenicity

**Fig. 3:**
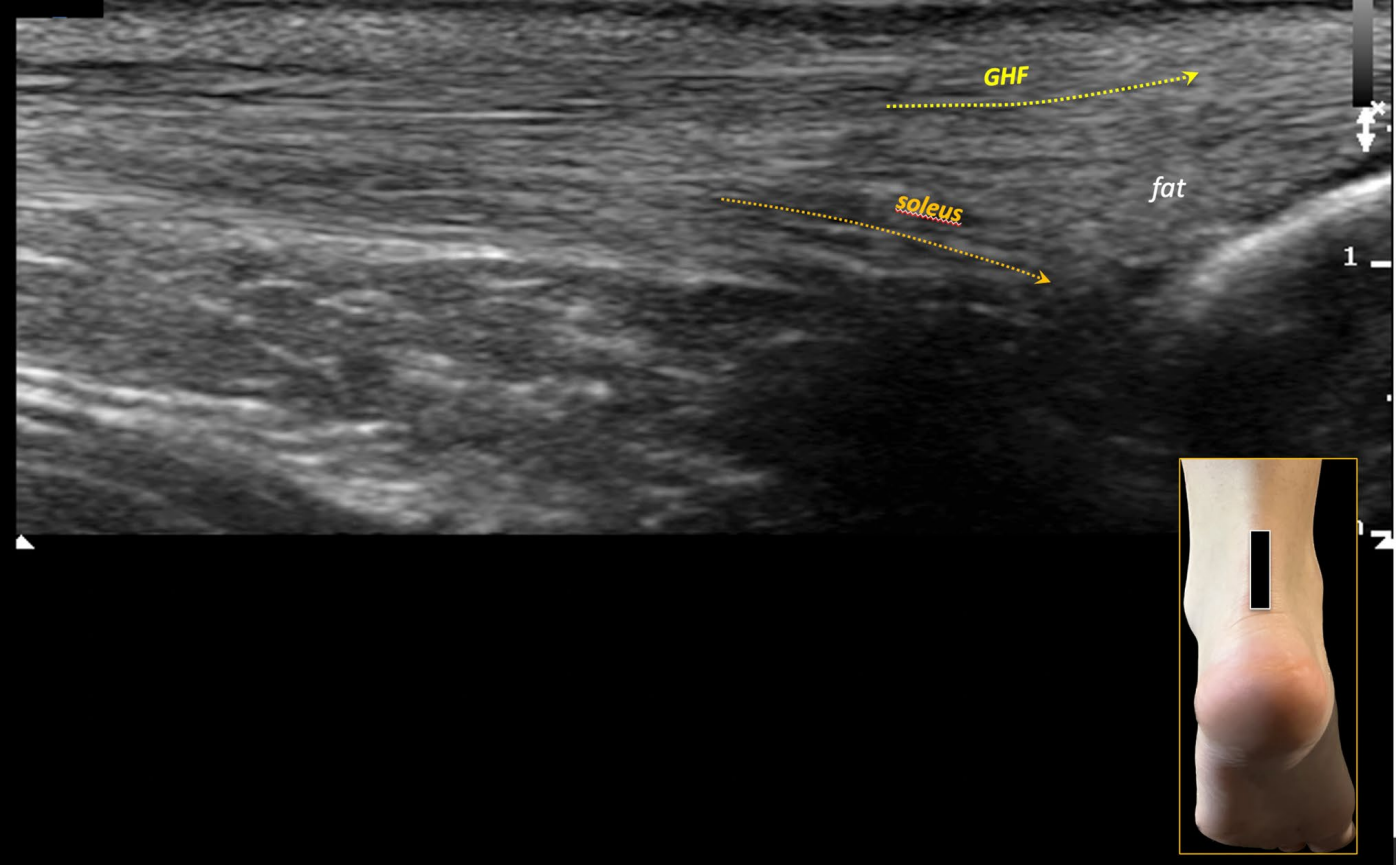
In long-axis scans, the two fascicles may occasionally have a distinct anatomical boundary due to separate insertions on the heel bone. In such cases, the soleus tendon is clearly separated from the gastrocnemius fascicle, inserting into the calcaneus with a fat triangle between the two components. This anatomical variant is present in monkeys. The figure shows divergent yellow dotted lines representing the soleus and gastrocnemius fascicles (GHF) inserting separately into the heel

**Fig. 4:**
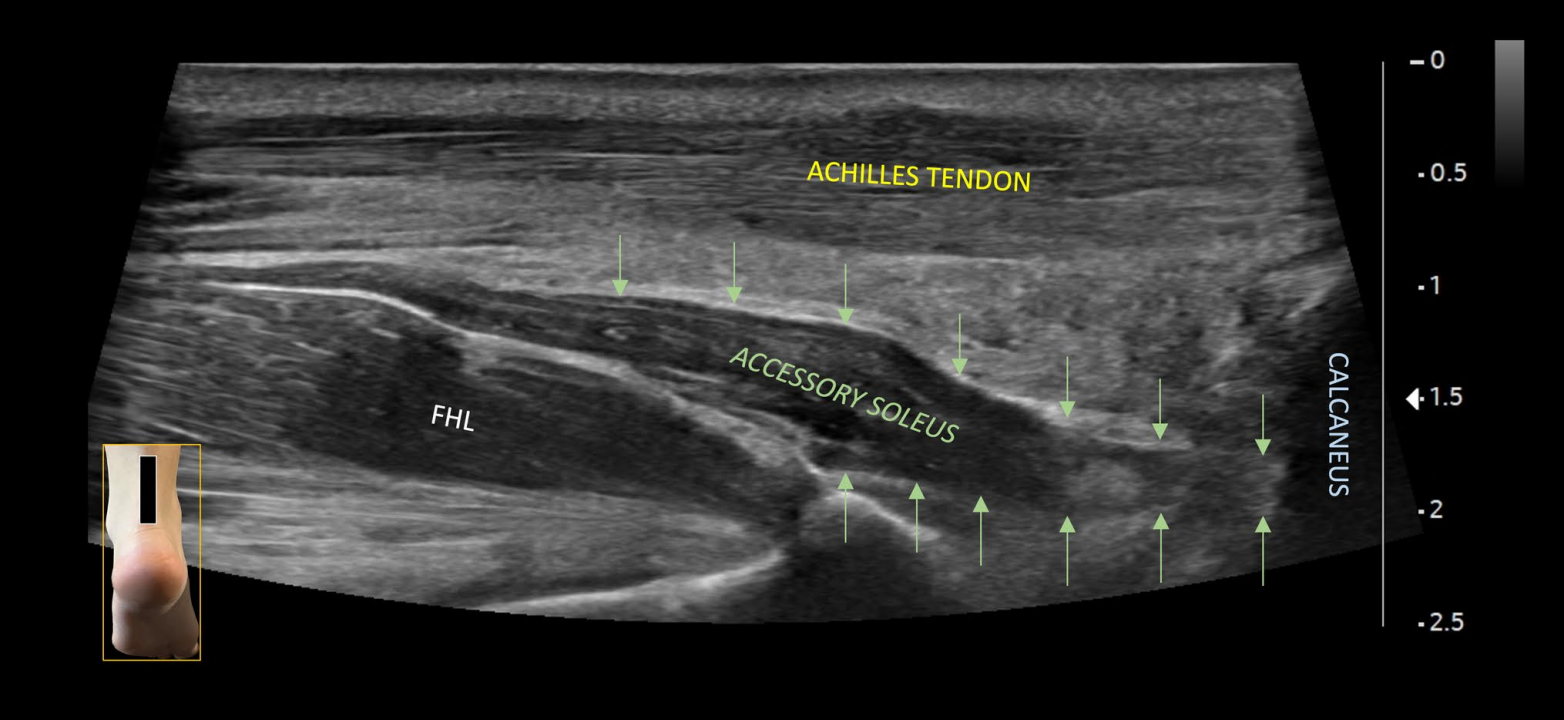
Long-axis scan of the Achilles tendon showing the accessory soleus muscle, which has its own muscle belly and tendon that inserts anteriorly and separately to the Achilles tendon enthesis. *FHL* Flexor Hallucis Longus. The green arrows indicate the belly and tendon of the accessory soleus, which inserts into the heel

- Superficial fascicles: The lateral and medial head of gastrocnemius fascicles (LMHGF) were consistently observed with relatively homogeneous echogenicity in the study population both generally exhibiting a slight hypoechogenicity, better seen in short axis.

- Deep fascicle (soleus): The deep component from the soleus shows generally increased echogenicity (Fig. 1). This fascicle shows variability in terms of separation from the above gastrocnemius fascicles, occasionally presenting with a distinct anatomical boundary that inserts separately on the calcaneal tuberosity (heel bone) [5–7] (Fig. 3): in these cases the soleus tendon is clearly separated from the superficial component of the gastrocnemius fascicles, inserting itself on the calcaneus with a fat triangle between the two components. This anatomical variant is present in monkeys [5]. The first identification of this variant led us to pay attention on the US evidence of the various fasciculations of the Achilles tendon. The accessory soleus muscle is not to confuse with this anatomical variant because it is a muscle with its own belly whose tendon is inserted anteriorly and separately to the enthesis of the Achilles tendon (Fig. 4).

The variation in echogenicity among the two superficial fascicles and the deep one depends on anisotropy for the different slight oblique orientation of these fascicles.

### Insertion and orientation

-The insertion points of the fascicles on the calcaneus show, as previously mentioned, that the lateral head of gastrocnemius, which has a biarticular insertion as the medial, inserts distally in the external part of the lateral calcaneal tuberosity while the medial head, adjacent to the latter, inserts slight medially and deeply respect the fascicle of the lateral head of gastrocnemius. Instead the soleus, that has a monoarticular insertion, inserts on the medial surface of the calcaneal tuberosity, but slightly more superiorly respect the fascicles of the gastrocnemius, and this may influence the overall stability of the tendon and susceptibility to medial injuries [3–11].

**Fig. 5:**
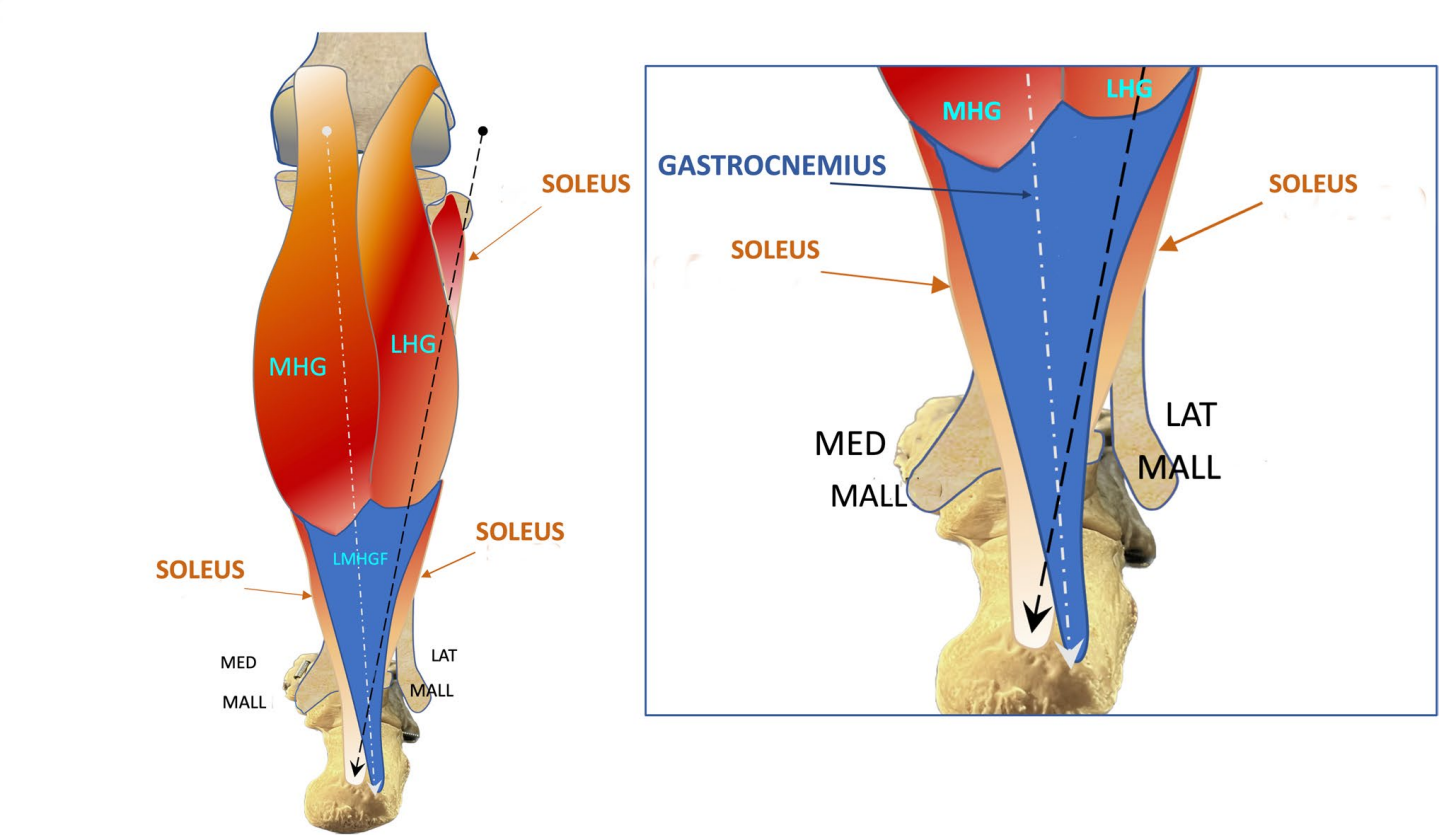
Schematic anatomical drawing showing the triceps surae fascicles crossing each other. The white dash-dotted line represents the direction of the gastrocnemius fascicles, while the black dashed line represents the direction of the soleus fascicle. The box on the right is an enlargement of the drawing. *MHG* Medial Head of Gastrocnemius, *LHG* Lateral Head of Gastrocnemius, *LMHGF* Lateral and Medial Head of Gastrocnemius, *LAT MALL* lateral malleolus, *MED MALL* medial malleolus

**Fig. 6:**
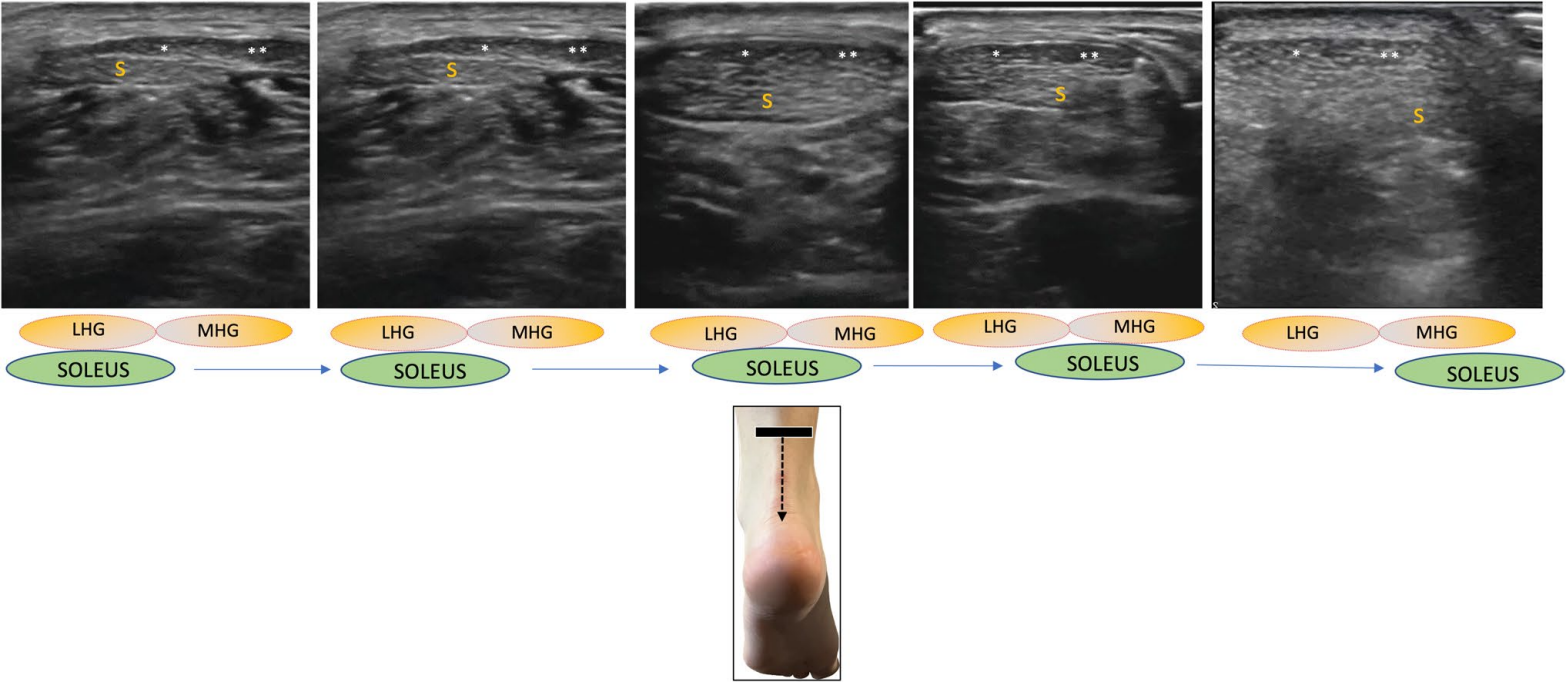
Short-axis scan of the three components of the left Achilles tendon in a dynamic sequence from top (starting with the first figure on the left) to bottom (last figure on the right). *S* soleus fascicle, *LHG* Lateral Head of Gastrocnemius, *MHG* Medial Head of Gastrocnemius

**Fig. 7:**
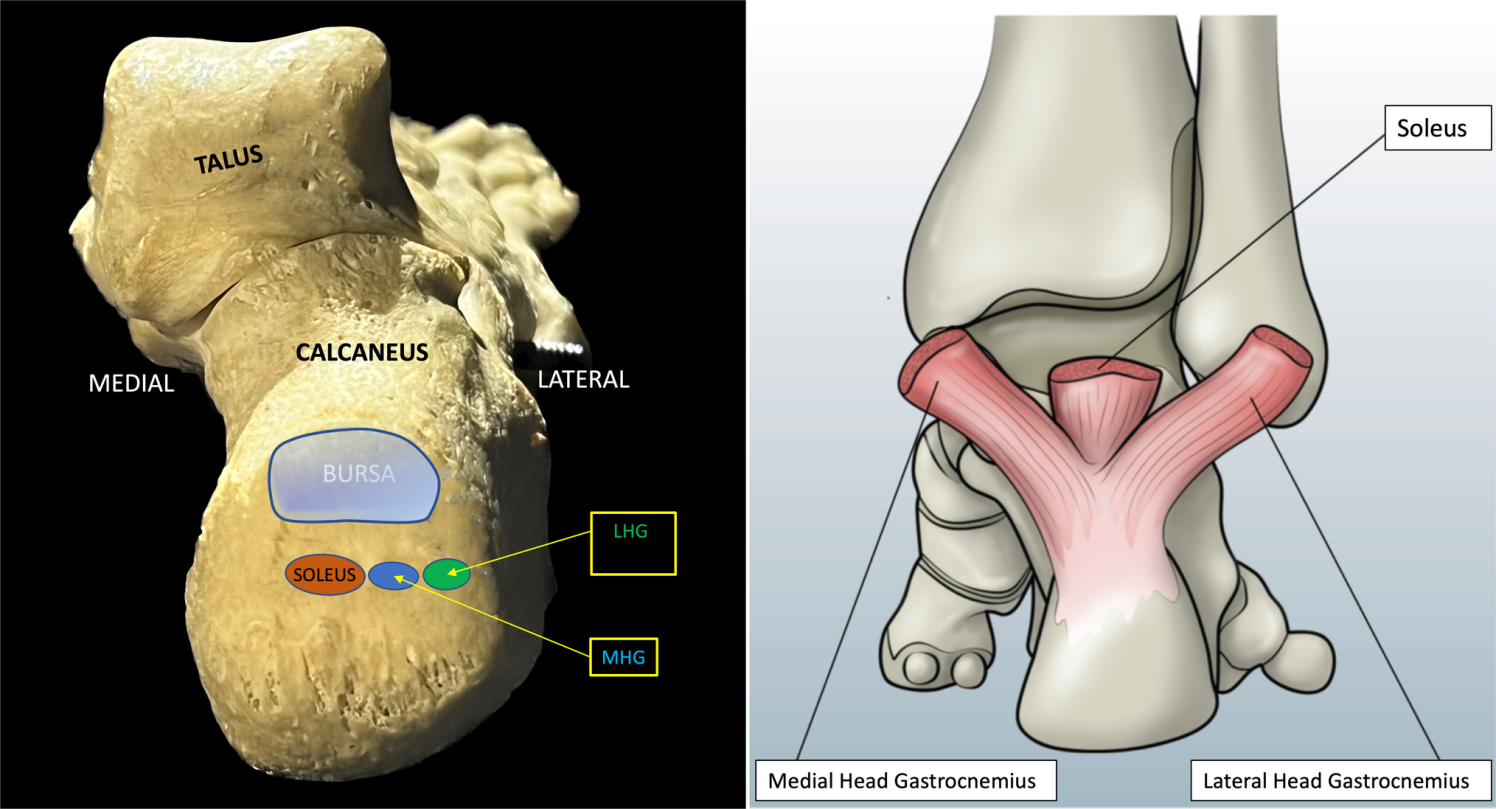
on the left, the insertions of the three fascicles into the calcaneal tuberosity on the calcaneus bone (see text). *LHG* Lateral Head of Gastrocnemius, *MHG* Medial Head of Gastrocnemius, *BURSA* retrocalcaneal bursa. On the right, schematic drawing of the three fascicles inserting into the heel

-The orientation of the fascicles at the insertion points may vary, with some fascicles showing a more vertical alignment and others a more angulated approach but it is important to point out that these fascicles cross each other in the distal portion of the Achilles tendon. The fascicles of LMHGF, proceeding from top to bottom, proceed from a situation of medial origin towards the lateral portion of the calcaneal tuberosity, while the soleus fascicle, starting proximally from a lateral position, moves to a medial situation with respect to the lateral and medial head of the gastrocnemius, crossing them (in front of them), inserting on the medial surface of the calcaneal tuberosity (Fig. 5, Fig. 6, Fig. 7A, Fig. 7B and Video1).

This orientation plays a crucial role in how forces are transmitted through the tendon and absorbed by the skeletal system [8, 9].

## US Findings

The visualization of distinct tripartite segmentation and fascicle orientation has significant implications for both clinical and athletic settings, particularly for approaches to diagnosing, treating, and preventing injuries.

The evidence of fasciculations of the Achilles tendon it becomes more evident in pathological conditions, expecially in the tendinopaty where it is well known that the sign of tendinosis is the fusiform thickening of the mid-portion of the Achilles tendon with increased thickness in both the longitudinal and transverse axes [10–14].

**Fig. 8:**
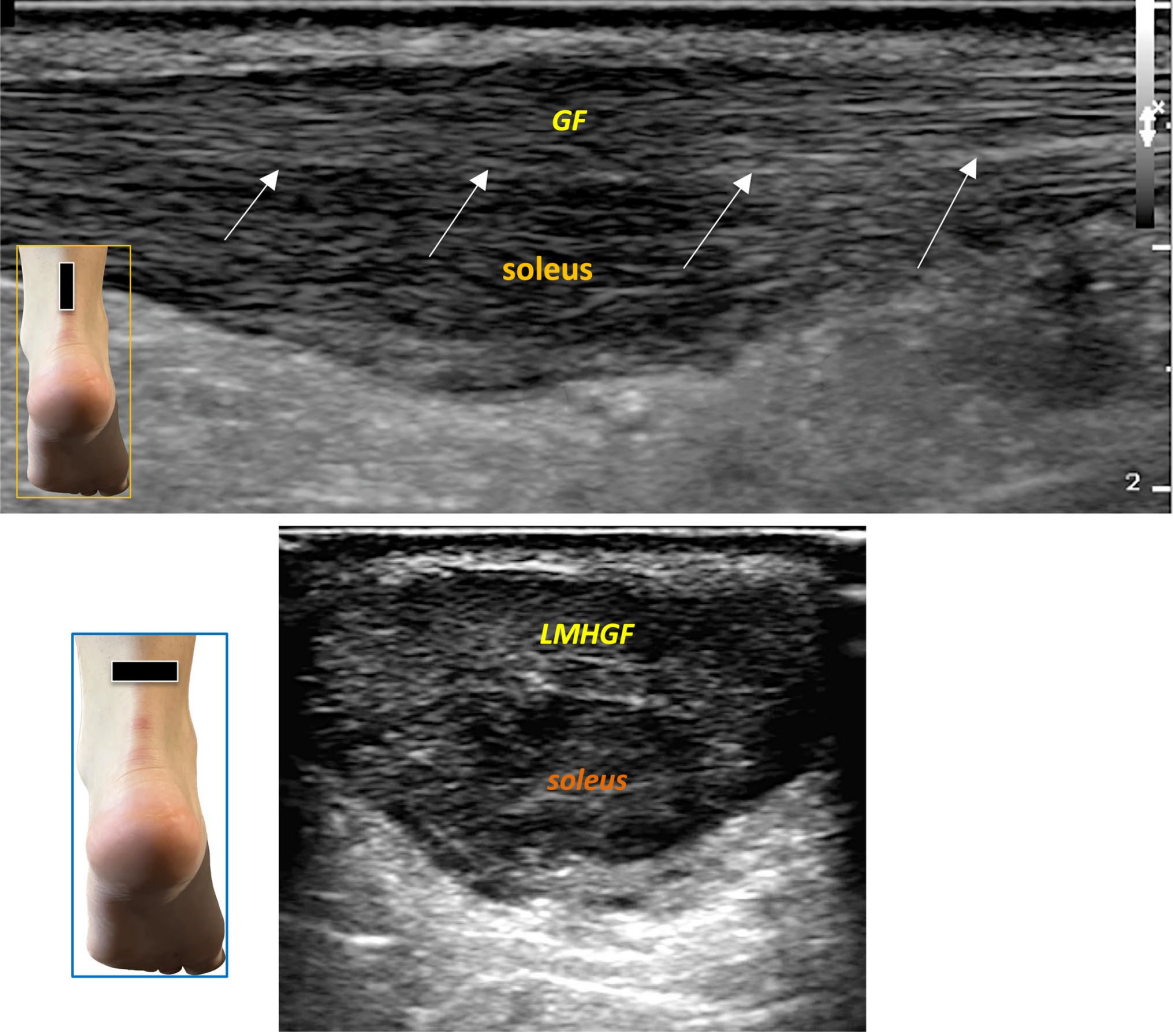
Evidence of fasciculations becomes more apparent in tendinopathy, where often only one partition (more frequently the soleus fascicle) is affected, while the remaining portion remains uninvolved, exhibiting a normal echopattern or mild pathological involvement. The figure shows a long-axis scan (above) of the Achilles tendon, predominantly affecting the soleus fascicle. *GF* Gastrocnemius fascicle. White arrows show the septum between the two components. The lower figure shows a short-axis scan: *LMHGF* Lateral and Medial Head of Gastrocnemius Fascicles

In these cases, it often occurs that one partition (more frequently the soleus fascicle) is affected, while the remaining portion remains uninvolved, exhibiting a normal echopattern or mild pathological involvement (Fig. 8).

**Fig. 9:**
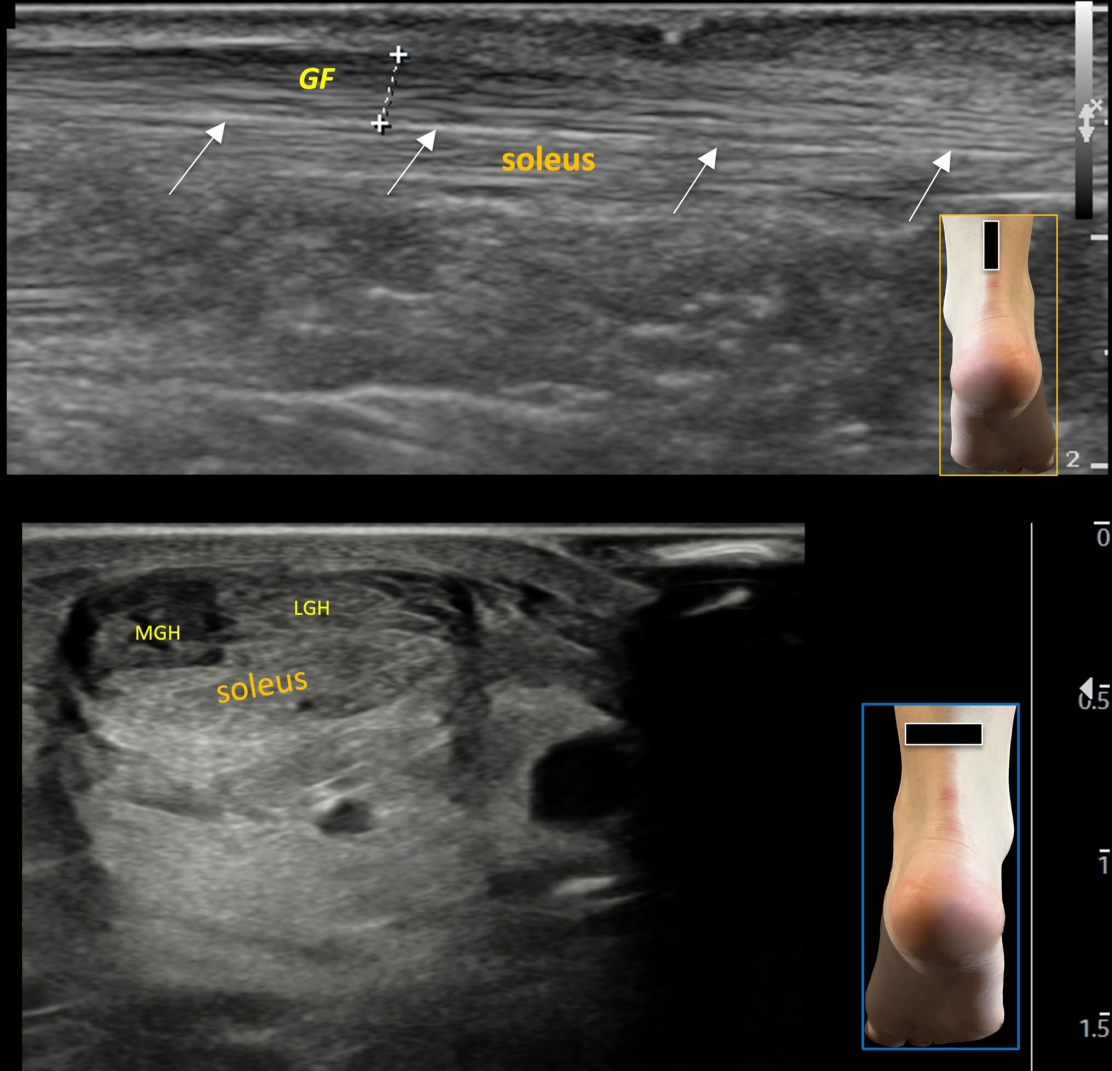
In tendinopathy, it is easier to distinguish the fascicles, identifying which partition is most affected. In longitudinal scans, the tendon appears bifasciculate, while in axial scans, a tripartite pattern is observed. In the upper figure, tendinopathy starts to affect the superficial layer (*GF* gastrocnemius fascicle). White arrows indicate the septum between the two components. The lower figure shows another case of focal tendinopathy affecting only the medial head of the gastrocnemius (MHG) in a short-axis scan, with the LHG (lateral head of gastrocnemius) appearing normal

In these conditions it is more easy to distinguisc correctly the fascicles, attributing to which fascicular partition the pathology is more affected, both in longitudinal scans where the tendon appears bifasciculate, or in axial scans where shows a tripartite pattern (Fig. 9).

It follows that the presence of crossed fasciculations of the Achilles tendon (an important factor in foot biomechanics) is particularly useful in the pathology of tendinosis, where precise identification of the diseased portion could guide a rehabilitative therapy aimed at one of the two affected muscle groups. [15, 16].

**Fig. 10:**
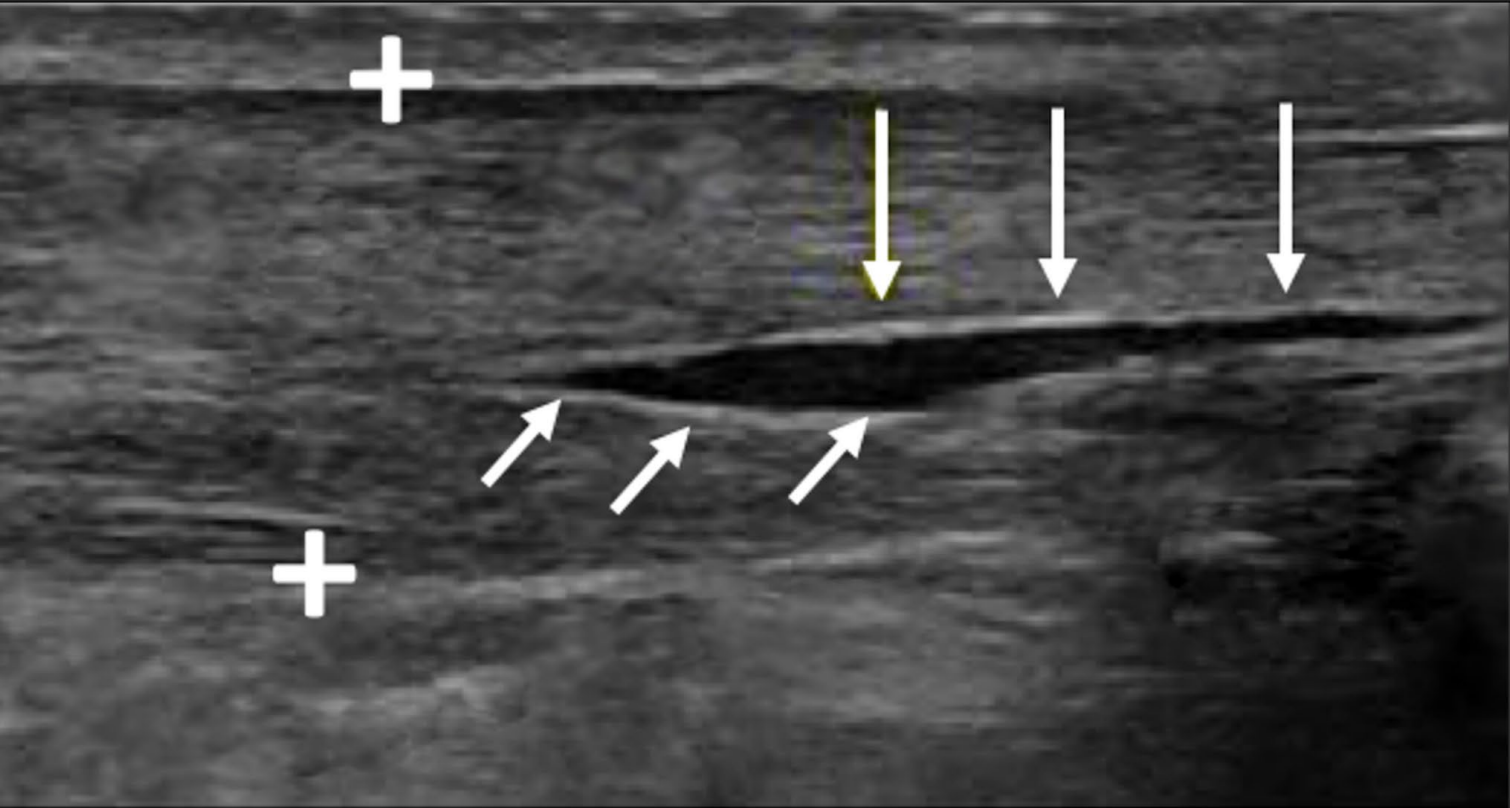
In cases of chronic tendinopathy, longitudinal fissures (intramural lesions) can also be identified, where partial tears localize in the septum between the gastrocnemius and soleus bundles. The Achilles tendon is shown between the calipers, with white arrows marking the fissures

Eventually, in case of chronic tendinopathy, it is possible to highlight longitudinal fissures (intramural lesions) where the partial tear is localized in the septum between the two bundles of the gastrocnemius and soleus (Fig. 10) [17, 18].

## Biomechanical perspectives and dynamic maneuvers

From a biomechanical perspective, the orientation of the tendon fascicles at their insertion points is particularly noteworthy. The alignment of these fibers can affect how forces are distributed across the tendon and subsequently to the calcaneus. Misalignment or anomalous insertion angles could result in abnormal stress distribution, potentially contributing to the development of overuse injuries. The findings from this study suggest that individual variations in fascicle orientation should be considered when assessing risk and designing rehabilitation protocols for tendon injuries.

Dynamic US examination of the Achilles tendon involves several maneuvers that assess the behavior of the tendon under stress and in motion in real time.

One such maneuver involves passive plantar and dorsiflexion. In this technique, the examiner passively moves the patient's foot through varying degrees of plantar and dorsiflexion. This movement helps visualize the behavior of the tendon under stress, allowing the examiner to detect any gaps or irregular motion that may indicate tears or ruptures.

Active motion is another aspect of dynamic assessment, where the patient actively moves the foot through plantar and dorsiflexion. Observing the tendon in motion during these movements can reveal more subtle pathology, such as partial or full-thickness tears, enabling easier differentiation of these conditions, which may not be as apparent on static images.

**Fig. 11:**
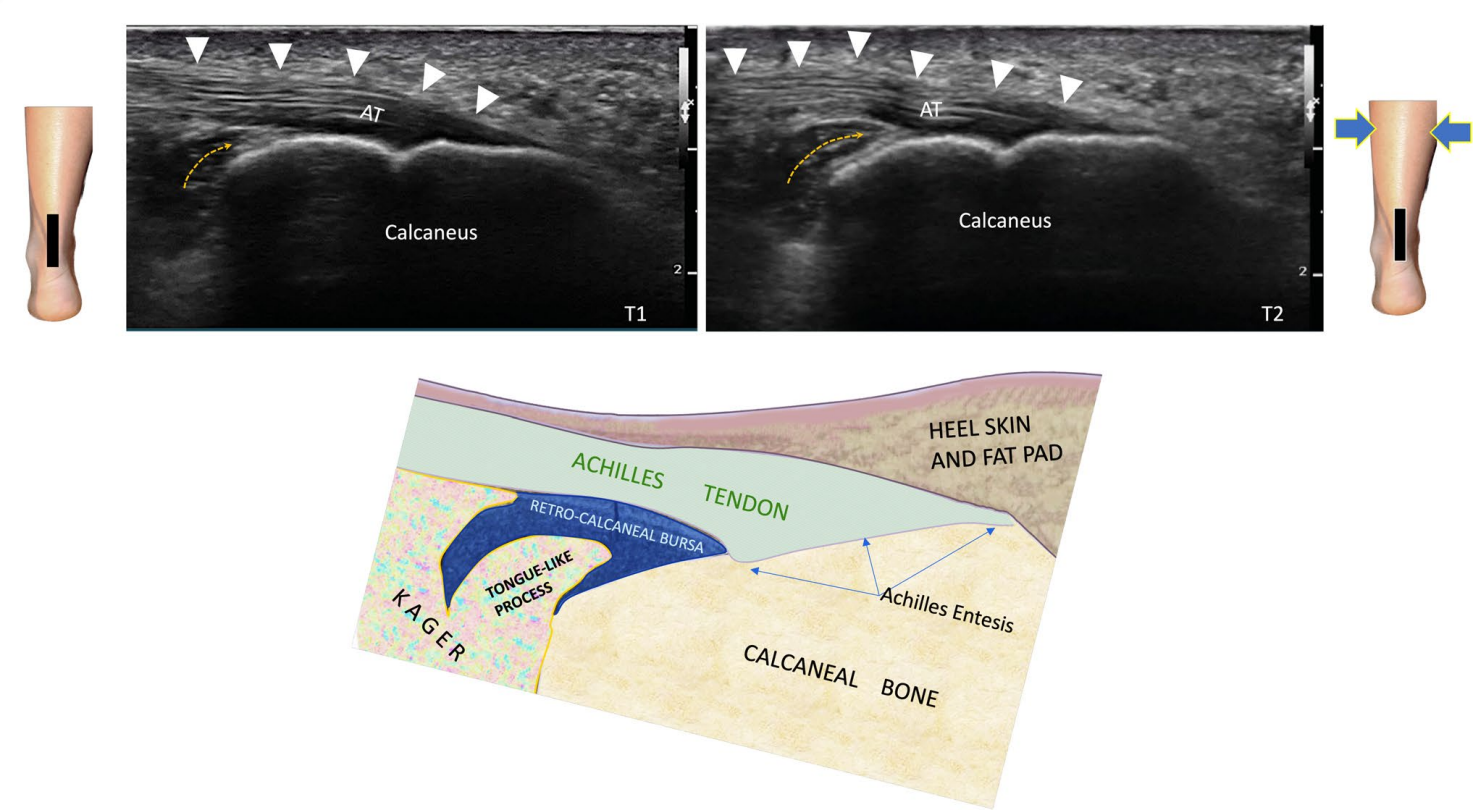
Dashed yellow arrows show the movement of the tongue-like process of the distal Kager’s fat pad (T1, T2) during the Thompson test. In this subject, the tendon elevates as the adipose process enters the bursal space. It has been hypothesized that this process may have a trophic and protective function for the overlying tendon. The schematic anatomical drawing below shows the position of Kager’s fat pad in relation to the Achilles tendon and the tongue-like process inside the bursa. *AT* Achilles tendon; the white arrowhead indicates the elevation of the tendon due to the movement of Kager's tongue-like process

Active muscle engagement provides a better picture of tendon function and any underlying problems. One particularly informative technique is the retrocalcaneal space squeeze and release. During this maneuver, the examiner holds the US transducer in a longitudinal plane on the posterior surface of the heel while gently pinching the retrocalcaneal soft tissues with the other hand; the same result can be obtained with the Thompson maneuver on the calf performed with the operator's free hand. This action displaces the bursal effusion into the deep retrocalcaneal bursa, improving visualization of the subsurface of the tendon [19, 20] with the possible presence of small tears. It is important to note that this maneuver also allows to observe the movement of the small adipose process which is part of the adipose triangle of Kager that presents a tongue-like shape, close to the entesis, that penetrates into the retrocalcaneal bursa during the flexion–extension of the foot (Fig. 11). It is recently hypothesized [21] that it would have a trophic and protective function of the overlying tendon. Therefore the Kager fat pad is an important anatomical landmark and its gliding smoothly in and out of the retrocalcaneal space without any difficulties, indicating normal function. In the Haglund's deformity the inflammation of Kager's adipose tissue almost ‘freezes’, greatly reducing the movement (Video2), presenting always an increased echogenicity and producing bursal effusion and suffering of the Achilles enthesis. This ‘squeenze’ or Thompson test helps to highlight any abnormalities that may not be visible during passive or active motion alone [12, 21–23].

In healthy tendons all these dynamic maneuvers reveal smooth, consistent motion of the Achilles tendon without significant gaps or irregularities.

**Fig. 12:**
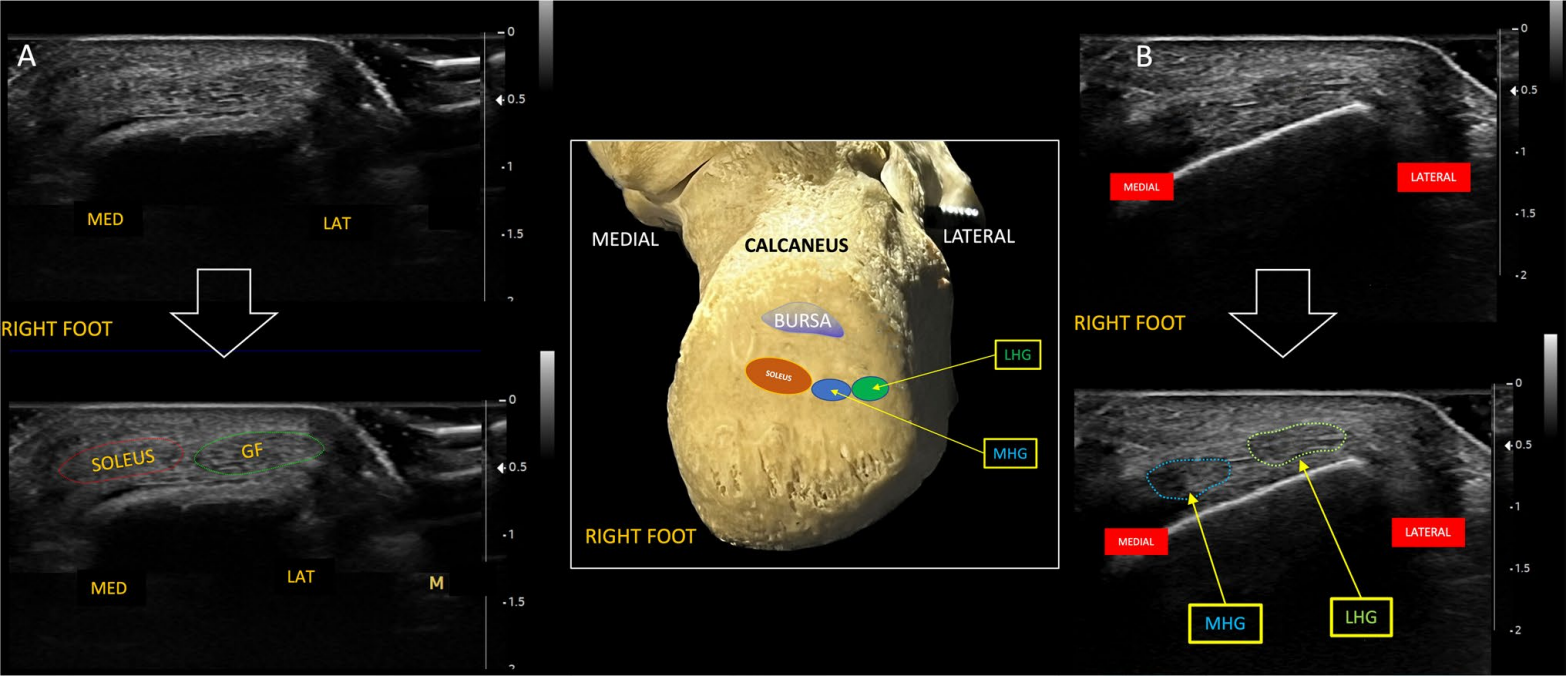
**A** short-axis scan of the fascicles *slightly* proximal to the enthesis, the soleus is directed on the medial surface of the calcaneal tuberosity (slightly superiorly) and both the two component of gastrocnemius are in lateral position. In this preinsertional scan the two fascicles of the gastrocnemius appear as a single fascicle. The figure below (great white arrow) is the same of the above one, with the indication for the two fascicles (*soleus* with red dots and *both the fascicles of gastrocnemius* with green dots). *MED* medial, *LAT* lateral. *GF* gastrocnemius fascicle. **B** short-axis scan at the insertion of the two fascicles of gastrocnemius, slight lateral and distally at the previous scan: the figure below (great white arrow) is the same of the above one, with the indication for the two fascicles of gastrocnemius: the lateral head of gastrocnemius (LHG, green dots) is inserted on the lateral surface of the calcaneal tuberosity and the medial head (MHG, blue dots), adjacent and medially to it. The soleus fascicle (not in figure) is inserted on the medial surface of the calcaneal tuberosity but slightly more superiorly. Between the two figures, there’s a schematic drawing (framed by a white line) of the three fascicles inserting into the heel

The study of the three fascicles in dynamic transverse cranio-caudal scans conclusively highlights that the two posterior fascicles of the gastrocnemius muscle intersect with the anterior fascicle of the soleus: the soleus component, from a position of lateral origin, moves inferiorly and medially up to the level of insertion (at the medial surface of the calcaneal tuberosity). The superficial component of the gastrocnemius with its two component (medial and lateral head), from a position of medial origin, moves laterally up to the insertion at the calcaneal tuberosity. The lateral head of gastrocnemius is therefore inserted on the lateral surface of the calcaneal tuberosity and the medial head, adjacent to it, medially to it. The soleus is inserted on the medial surface of the calcaneal tuberosity, but slightly more superiorly, and all this is in agreement with the most recent anatomical study by Ballal [3] (Fig. 12). Although Ballal and Szaro [2, 3] and other anatomists have described more than three fascicles, with ultrasound we are able to identify only three of them.

*Relation to Previous Studies*: The first anatomist who described fasciculations of the Achilles tendon was, in 1944, Frederic Wood-Jones [1]. Recently anatomical studies of Ballal and Szaro [2, 3] deepened the study highlighting more fasciculations of the tendon. From a recent and accurate research in diagnostic imaging literature we found a first description by Bertolotto (1995) (10) that, using a high frequency experimental probe (a 10–15 MHz mechanical-sector probes with a built-in water path), described only a double component, but this report has not received subsequent feedback, thus remaining neglected to date. In 2015 a pattern of fascicular involvement in midportion of achilles tendinopathy at US was reported by Counsel (11) but the anatomical location of the fascicles does not coincide with our report. To our knowledge after these observations no other author has described the fascicles of the Achilleus with US. The detailed US approach used in this study highlights these structural variations, which are often overlooked but may hold the key to understanding why some individuals are more susceptible to tendon pathology than others.

*Future Research Directions*: these anathomical considerations opens several avenues for future research. Longitudinal studies could explore how echogenicity and fascicle orientation change with age, activity level, and in response to injury. Additionally, biomechanical modeling studies could use the detailed anatomical data provided here to simulate different loading conditions on the Achilles tendon, helping to predict areas of potential stress concentration in various physical activities.

*Therapeutic Implications*: From a therapeutic perspective, the insights gained from this study could revolutionize the approach to rehabilitation following Achilles tendon injuries. By understanding the specific structural characteristics of an individual’s Achilles tendon, and recognizing that the gastrocnemius has a biarticular insertion as opposed to the monoarticular soleus, rehabilitation specialists can tailor exercises to optimize load distribution and facilitate tendon healing while minimizing the risk of re-injury. Additionally, preventive measures could be more effectively designed by identifying at-risk individuals based on the US characteristics of their tendons. The advanced US technique employed in this study has provided new dimensions to the anatomical and biomechanical understanding of the Achilles tendon. These insights not only aid in the clinical management of tendon disorders, but also contribute to a deeper scientific understanding of tendon dynamics, which is essential to advance preventive and therapeutic strategies.

*Conclusion*: This essay offers a detailed visual and textual exploration of the anatomy of the Achilles tendon and its fascicles with important clinical implications. The findings highlight the need for advanced imaging techniques in both research settings and clinical practice, which could potentially lead to improved diagnostic and therapeutic strategies.

## Supplementary Information

Below is the link to the electronic supplementary material.

Supplementary file1 (MP4 3099 KB)
Video 1: Short-axis scan of the three components of the left Achilles tendon in a dynamic sequence from top (starting with the first figure on the left) to bottom (last figure on the right). S: soleus fascicle; LHG: Lateral Head of Gastrocnemius; MHG: Medial Head of Gastrocnemius

Supplementary file2 (MP4 5300 KB)
Video 2: In Haglund’s deformity, the inflammation of Kager's fat pad leads to a significant reduction in its mobility, almost 'frozen'. This condition is characterized by increased echogenicity, bursal effusion, and stress at the Achilles enthesis. The video demonstrates the small tongue-like extension of Kager's fat pad in Haglund’s deformity on the right side, showing increased echogenicity and reduced mobility compared to the healthy contralateral side (on the left)

## Data Availability

Not applicable.
